# The Knowledge, Practice and Attitudes of Nurses Regarding Physical Restraint: Survey Results from Psychiatric Inpatient Settings

**DOI:** 10.3390/ijerph18136747

**Published:** 2021-06-23

**Authors:** Tsz-Kai Lee, Maritta Välimäki, Tella Lantta

**Affiliations:** 1 Department of Psychiatry, Tai Po Hospital, Tai Po, New Territories, Hong Kong, China; ltk975@ha.org.hk; 2 School of Nursing, the Hong Kong Polytechnic University, Kowloon, Hung Hom, Hong Kong, China; 3 Xiangya Nursing School, Central South University, Changsha 410013, China; 4 Department of Nursing Science, University of Turku, 20014 Turku, Finland; tejela@utu.fi

**Keywords:** physical restraint, nursing knowledge, nursing practice, nursing attitudes

## Abstract

There is a considerable amount of literature describing how nurses’ knowledge contributes to their attitudes and practices related to patient physical restraint. However, whether or not there have been any improvements in nurses’ knowledge levels, attitudes or practices regarding physical restraint during the past few years is unknown. A survey was conducted on nurses (*n =* 133) in one psychiatric hospital in Hong Kong (*n =* 98, response rate = 74%). The data were analyzed using independent t-tests, ANOVA, a Mann–Whitney U test, a Kruskal–Wallis test and Spearman’s rho. In general, nurses had good restraint-related knowledge with satisfactory attitudes and practices, although their knowledge levels, attitudes, and practices regarding restraint varied. Having a higher age, seniority, and education level contributed to a higher restraint-related knowledge level. Male nurses demonstrated more desirable practices (i.e., care of restrained patients), while nurses with a higher education level were more likely to avoid restraint. Nurses’ restraint-related knowledge positively correlated with restraint practices. Although nurses’ knowledge levels, attitudes, and practices regarding restraint were found to be satisfactory, more training efforts should focus on young nurses working in psychiatric settings with less work experience and lower education levels. As some nurses seem to favor the use of restraint with limited reflection, more studies are needed to verify nurses’ emotions and how their emotions influence the use of restrictive practices.

## 1. Introduction

Patient physical restraint is a controversial topic in healthcare services [1]. Physical restraint, such as magnetic restraint, belts or manual restraint, is used to protect patients from self-harm and to keep them from harming others [2]. However, various adverse effects for patients have been reported, such as psychological distress, affected physical health condition [3] and even death [4]. It has also been reported that the use of restraints exposes staff to occupational hazards [5] and psychological burden [6]. Further, a systematic review by [7] estimated that economic costs for using force, manual restraint and seclusion can total EUR 28,518 annually for one ward.

In a review conducted by [8], the authors revealed that restraint can be a form of abuse when it is inappropriately used, and can result in fear, neglect, and failure to use de-escalation techniques. Therefore, more critical attitudes towards these restrictions are needed [9]. Staff members’ abilities to reflect on their own attitudes, emotions and actions should also be promoted in order to reduce coercive practices [10], which should be used as a last resort when other less restrictive methods are exhausted [11]. Indeed, intervention approaches such as the Safewards model have been shown to increase meaningful activities on wards and thereby decrease the use of coercive practices [12]. Such approaches involve a package of agitation management strategies, including expanding de-escalation skills among ward staff; regular patient meetings to facilitate inter-patient support; having a structured, personal and innocuous information flow between staff and patients; and displaying positive messages about the ward from discharged patients [12]. Other good examples include patient involvement in aggression management [13] and recovery-oriented programs with anger management technique training for the staff and patients involved [14].

Despite the wide range of existing international studies regarding the use of physical restraint, the literature in this field is still not globally well-targeted. Still, the topic is important because restraint is currently a common practice in Asian psychiatric hospitals. In mainland China the prevalence of using mechanical restraints in psychiatric care has been reported to vary between 27.2% (*n =* 1364 patients) [15] and 51.3% (*n =* 160 patients) [16]. In one study carried out in Hong Kong, 39.7% (*n =* 335) of patients were restrained within the first week of admission [17]. In Taiwan, 29.5% (*n =* 59) of patients visiting psychiatric emergency care were restrained during their care episode [18]. A review by [19] including studies from Chinese language databases concluded that the frequency of restraint is higher in China compared to average numbers globally. From the patient’s perspective, the use of restraints was perceived negatively: over half (61.2%) of psychiatric inpatients surveyed in Hong Kong (*n =* 129) reported traumatic experiences due to witnessing another patient being taken down and 41.1% of that group reported traumatic experiences due to witnessing another patient being put in restraints of any kind [20].

Nurses are the primary decision makers who apply restraints in psychiatric care settings [21]. Although nurses were found to view the use of restraints as an inevitable intervention in psychiatric inpatient settings, necessary for calming patients, previous studies have reported that nurses have acknowledged that the intervention should be a “last resort” to use only after all the less coercive alternatives have been tried [6]. However, it has been assumed that the knowledge and attitudes among staff may directly or indirectly affect restraint practices and thus the likelihood for initiating restraints [22]. A systematic review by [23] concluded that, during the last two decades, staff’s attitudes towards coercion have changed from seeing it as part of therapeutic care towards viewing it as a matter of safety. Indeed, this may be the case in countries where significant efforts have been made to diminish the use of coercion, especially restraint [24,25]. Still, outside Western countries, studies report only moderate knowledge and attitudes towards coercion and the use of physical restraints [26,27].

To better understand the current situation in Hong Kong and to allow comparability to other countries and previous situations in Hong Kong [28], we conducted a survey using the Physical Restraint Questionnaire (PRQ) [29]. The PRQ is an instrument to measure nurses’ knowledge, practice, and attitudes regarding physical restraint. This instrument and its various language versions have been used in inpatient psychiatric settings in Hong Kong [30], Finland [31], India [32], Malaysia [33], Saudi Arabia [26], Sudan [11], and Turkey [34,35,36].

The PRQ has been previously used for both evaluating effectiveness of interventions and for cross-sectional study purposes. Results from two trials with continuing education programs offered contradictory results using the PRQ. An eLearning course for nurses did not have long or short-term effects on nurses’ knowledge, attitudes or practices regarding the use of physical restraints [31], while another study with group-based psychoeducation for nurses had positive short-term effects on nurses’ knowledge, attitudes, and practices [35]. Mixed results have also been found in cross-sectional studies. Levels of knowledge, attitudes and practices have varied between studies from moderate to excellent [33,34]. For instance, clear deficits have been detected in nurses’ knowledge about treatment complications [34] and alternatives [33] related to physical restraints. On the other hand, results regarding factors associated with nurses’ knowledge, attitudes, and practices were partially inconsistent between studies. Nurses’ gender, level of education, years of work experience and type of working unit seem to impact knowledge, attitudes and practices regarding the use of restraints, although findings appear to vary between countries. One previous study was conducted in local psychogeriatric wards in Hong Kong [30] with a small sample size (*n =* 42 nurses). The study’s findings were inconsistent with several international studies [37,38] in terms of nurses’ emotions towards restraint; more than half (*n =* 15, 53%) of the nurses had no ethical dilemma regarding restraints [30]. In this study, we therefore describe the current state of the knowledge, practices and attitudes among nurses about physical restraint and the associated factors at a psychiatric inpatient hospital in Hong Kong, and compare them to previous local and international findings. We aim to identify potential gaps in current local practices in Hong Kong to identify where targeted interventions could possibly be offered to improve nurses’ know-how related to psychiatric inpatient care.

## 2. Materials and Methods

### 2.1. Study Design

A cross-sectional design was adopted for this study.

### 2.2. Setting

The study was conducted in one psychiatric department at a hospital in Hong Kong. The hospital provides psychiatric inpatient and outpatient services (day hospital and community psychiatric services) to people living in one cluster with a population of less than 2 million [39]. The study was conducted on 8 inpatient wards (4 male and 4 female wards), of which all used restraint intervention. The wards specialized in general adult psychiatry, learning disabilities, substance abuse, and psychogeriatrics. Patients were admitted on voluntarily or compulsorily bases. At the time of data collection in December 2016, the average patient capacity was 45 beds per ward, and the length of stay was approximately 36 days.

### 2.3. Study Population and Eligibility Criteria

Our target population was nursing staff working in inpatient psychiatric care. The current size of the psychiatric nursing staff population in Hong Kong is 3266 registered nurses (psychiatric) and 1637 enrolled nurses (psychiatric) [40]. The Hospital Authority manages 43 public hospitals and institutions, 49 specialist outpatient clinics and 73 general outpatient clinics in Hong Kong. These organizations are divided into seven hospital clusters based on their locations. The aim of the hospital clusters is to ensure that patients receive a continuum of high-quality care within the same geographical setting and throughout their episode of illness from the acute phase through convalescence, rehabilitation, and community after-care [41].

In our study of wards in December 2016, there were a total of 157 nurses (1 department operational manager, 7 ward managers) and other included nurses were advanced practices nurses, registered nurses and enrolled nurses) with a high school diploma or below, a bachelor’s degree, a master’s degree or a PhD.

The inclusion criterion for the participants was that they were qualified nurses (i.e., nurses enlisted in the Nursing Council of Hong Kong) [42] who provided direct patient care and who worked in inpatient settings during the time of data collection. The exclusion criteria were if they were nursing students, nurses on any kind of leave during the data collection period, or nurses working in outpatient settings.

### 2.4. Instruments and Variables

Background information was collected on gender (male/female), years of experience as a qualified nurse, educational level (high school diploma or below/bachelor’s/master’s degree or above) and exposure to management of violence (MOV) training (yes/no). In Hong Kong, in-service training for management of violence in all psychiatric hospitals consists of three levels of training: the first and second levels involve basic workplace violence awareness with de-escalation techniques and break-away techniques respectively, and they are mandatory for all qualified psychiatric nurses, whereas the third level deals with control and restraint techniques for patients with agitation/aggression, using a team approach [43].

The Physical Restraint Questionnaire (PRQ) [29] was used to measure the nurses’ knowledge, practices and attitudes regarding physical restraint. A physical restraint is defined by [29] as “any article, device, or garment that interferes with a person’s free movement and secures him or her to a bed or chair” (p. 345). The PRQ was selected because it allows international comparisons, its content is suitable in the Hong Kong practices, and its validity and reliability have been reported to be good [44].

The PRQ includes three sections. First, the Knowledge section consists of 18 statements using a 3-point Likert scale (“true,” “false,” “not sure”). Every correct answer gets 1 point, while indicating “not sure” or providing a false answer merits 0 points. The total score can range from 0 to 18; a higher score indicates a higher number of correctly answered items [45]. The intraclass correlation coefficient has been established to be 0.85 [44] and the Cronbach’s alpha to be 0.65 [46]. Second, the Practice section consists of 18 statements measured with a 3-point Likert scale (“Always,” “Sometimes,” “Never”) [45]. This section concerns the nursing care prior to and during the use of restraints. In this section, 4 statements are negatively phrased, and the ratings are reverse-scored (“Never,” “Sometimes,” “Always”). A total score of 18 represents the least desirable practice and 54 the most desirable practice. The intraclass correlation coefficient has been established to be 0.99 [44] and the Cronbach’s alpha to be 0.94 [46]. Third, the Attitudes section consists of 12 items measured with a 3-point Likert scale (“Agree,” “Neutral,” “Disagree”) [45]. Again, negatively phrased items are reverse-scored. The total score ranges from 12 to 36. The lower the score, the less desirable the attitudes among nurses towards restraint. The intraclass correlation coefficient was established to be 0.84 [44] and the Cronbach’s alpha to be 0.61 [46].

### 2.5. Data Collection and Sample Size

The data were collected between 12 December 2016 and 2 January 2017. First, ward managers of the 8 wards were contacted one week before the data collection period to confirm the number of nurses for which to prepare the questionnaires. They were provided with an explanation letter for the study and asked to encourage staff participation and facilitate opportunities for them to complete the questionnaires, so as to boost the response rate. Second, on the first day of data collection, the questionnaires were delivered by the researcher to the clerical staff of each ward, and subsequently distributed to the participants in their personal mailboxes. A temporary drop-box was placed on each ward to facilitate the collection. Third, the questionnaires were collected by the researcher in person on the final day of data collection.

The sample size required to represent the population was calculated to be 96 with a margin of error of 10% and a confidence level of 95% [47]. To achieve the sample size needed, convenience sampling was used. All nurses fulfilling the eligibility criteria were invited to participate in the study.

To manage missing data, list-wise deletion (excluding any questionnaires returned with missing values) [48] was used, since less than 5% of the questionnaires had missing data [49]. Answers to negatively phrased statements were reversed to be congruent with the answers of the other items.

Due to the small sample size, personal data of the respondents were recategorized for a more relevant statistical analysis, and the data were presented using descriptive statistics (standard deviation, frequencies, percentages, mean, median, frequencies and percentages). To generate the mean total score of a particular section (i.e., Knowledge, Practices, Attitudes), the ratings of a participant were first summed up to calculate a total score. After that, the calculation was repeated to generate an array of total scores for all the participants regarding each section. All of these total scores were summed up to generate a mean total score [50].

Nurses’ responses in the Practice and Attitudes sections were also recategorized. In the Practice section, the 3-point scale (“Always,” “Sometimes,” “Never”) was recategorized into 2 categories (“Always,” “Sometimes/Never”): responses in the negatively phrased statements (e.g., “All disoriented acute care patients should be restrained”) were recategorized from “Never,” “Sometimes,” “Always” to be “Never,” “Sometimes/Always.” Similar recategorizations were done in the Attitudes section: responses were recategorized from a 3-point scale to be “Agree” or “Neutral/Disagree,” while responses in the negatively phrased statements were grouped as either “Disagree” or “Neutral/Agree.”

### 2.6. Statistical Methods

Independent t-tests [49] or ANOVA were used for the total score(s) that were normally distributed. In cases of non-normal distribution of the scales, a Mann–Whitney U-test (median test) or Kruskal–Wallis test was used to compare possible differences in scores between the sub-groups (e.g., males vs. females) [49,51]. An ANOVA or Kruskal–Wallis test was used to describe any group differences, and a post-hoc analysis was used to identify in which specific groups a difference could be found [49] by using the Bonferroni test for ANOVA [49] and the Dunn’s test for Kruskal–Wallis [52]. In addition, Spearman’s rho was used [50] to describe correlation between the knowledge, practice and attitudes scores.

All analyses were performed using SPSS 23 (IBM Corp. IBM SPSS Statistics for Windows, Version 23.0; Armonk, NY, USA) [53], and the level of statistical significance was set to *p* < 0.05 [51].

### 2.7. Ethical Considerations

Ethical approvals were granted by the local university human ethics committee (HSEARS20160923001) and at the hospital ethics committee (CRE-2016.536). An information letter of the study was given to the participants as the cover of the questionnaire to explain the study’s purpose, procedure, voluntary participation as informed consent (returned and completed questionnaire was implied to be consent to participate), and privacy issues. Anonymity and confidentiality were ensured by coding all completed questionnaires [54].

## 3. Results

### 3.1. Participants

Out of our total study population (*n =* 157), 133 were eligible to participate in the study. Those who were excluded in the target population worked in outpatient settings. We received 98 completed questionnaires (response rate 74%).

### 3.2. Descriptive Data

Out of 98 nurses, males were in a slight majority (53.1% vs. 46.9%). Ages ranged from 25 to 55 years (Mdn (median) *=* 30, inter-quartile range = 11). About three-quarters (*n =* 70, 71.4%) had a nursing education level higher than a bachelor’s degree, and half (*n =* 49, 50%) had 5 or more years of nursing experience. All of them (100%) had completed mandatory in-service training for the management of violence (see Table 1).

### 3.3. Level of Knowledge

Most nurses (89.8%, *n =* 98) agreed with the statement “a physical restraint requires a physician’s order.” Three-quarters (75.5%) believed that good alternatives to restraints exist. However, only one-quarter (26.5%) agreed that “Restraints should be released every 2 h if the patient is awake,” and about one-fifth (22.6%) agreed that restraining a patient while lying flat on a bed should be avoided because of danger of choking (See Table 2).

Nurses’ scores representing their knowledge levels of restraint ranged from 3 to 17 (Mdn = 12; Interquartile Range = 3), while the possible maximum score was 18. Each nurse’s demographic information was compared with their knowledge level of restraint. Statistically significant results were found regarding age, experience and education. First, nurses who were less than 30 years old had lower knowledge levels than those aged 30 or over (Mdn = 11 vs. 12, U = 725.5, *p =* 0.001). Second, nurses with 5 to 9 years of clinical experience had the highest knowledge levels among the 3 experience categories (χ^2^ (2), *n =* 98) = 11.69, *p =* 0.003). Dunn’s post-hoc test indicated that there was a significant difference between nurses with experience of 5 to 9 years and those with 4 years or less (Mdn = 13 vs. 11, *p =* 0.003). Third, those who had a master’s degree or higher had the highest knowledge levels among the 3 education categories (χ^2^ (2), *n =* 98) = 14.86, *p =* 0.001). Dunn’s test indicated that nurses with a master’s degree or above had significantly higher knowledge levels than those with a bachelor’s degree (Mdn = 14 vs 12, *p* = 0.003) and those with a high school diploma or below (Mdn = 14 vs. 11, *p =* 0.001) (see Table 3).

### 3.4. Nursing Care of Patients Immediately before or during Restraint

The vast majority of the nurses (94.9%, *n =* 98) indicated that they would always try alternative measures before applying restraints. Many of them (89.8%) would respond to a restrained patient’s call for help as soon as possible and explain the reason of restraints to patients. However, many nurses (92.8%) agreed that restraint could be necessary for the prevention of self-harm, and they reported that they would routinely apply restraints on disoriented (67.3%) patients and confused patients with arterial or venous lines (68.4%). Few (39.8%) disagreed that more patients would be restrained during times of limited manpower (see Table 4).

Nurses’ scores in the Practice section ranged from 38 to 53 (Mdn = 46; Interquartile range = 4), while the possible maximum score was 54. When each nurse’s demographic information was compared with their restraint practice, a statistically significant difference was found in gender. The restraint practices of male nurses were more desirable than those of female nurses (Mdn = 48 vs 46, U = 913, *p =* 0.042) (See Table 5).

### 3.5. Attitudes towards the Use of Restraint

Most nurses (70.4%) believed that they had the right to refuse placing patients in restraint, and the majority of them (87.8%) believed that it is important to let the patient know they care about them. In contrast, few nurses (13.3%) felt guilty about applying restraints, while 15.3% agreed that patients become more confused after being restrained. One-quarter (25.5%) considered that patients’ relatives had the right to refuse restraint. Further, about one-third (31.6%) had negative feelings if a patient got more upset after being restrained (see Table 6).

Nurses’ scores representing their attitudes towards restraint ranged from 18 to 36 (M = 25.9, SD = 3.6), while the possible maximum score was 36. When nurses’ demographic information was compared with their attitudes towards restraint, a statistically significant result was found with regard to education. Nurses with a master’s degree or higher had the most desirable attitudes (F(2.95) = 9.67, *p* < 0.001). Bonferroni’s post-hoc test further indicated that nurses with a master’s degree or higher had significantly more desirable attitudes than those with bachelor’s level (M = 28.59 vs. 25.19, *p* < 0.001) or high school level education or below (M = 28.59 vs. 24.89, *p =* 0.001) (see Table 7).

### 3.6. Correlations between the Total Scores of PRQ Sections

Possible correlations between total scores of knowledge, practice and attitudes were analyzed by Spearman’s rho. There was a statistically significant positive moderate correlation between knowledge and practice (rs = 0.38, *p* < 0.001), in which a correlation coefficient between 0.3 and 0.5 indicates a moderate relationship [50]. The higher knowledge level regarding restraint the nurse had, the more desirable their practice was found to be. However, neither knowledge and attitudes, nor attitudes and practice were found to have significant correlations (see Table 8).

## 4. Discussion

Findings of this study showed that nurses had good restraint-related knowledge with satisfactory attitudes and practices. As in previous studies, more senior and older nurses had good knowledge levels compared to less experienced and younger nurses. On the other hand, only one-quarter of nurses agreed that restraints should be released every 2 h. Nurses’ responses may reflect local restraint practice guidelines, which were announced by the Hospital Authority Head Office [55], causing nurses to prefer an even shorter intended restraint duration (less than 2 h). Nevertheless, the shorter the physical restraint period, the less likely adverse physical outcomes will be, such as asphyxia, laceration or even death [56]. However, similar to previous studies [26,30,32], areas of misconception among nurses were found. For example, participants indicated that it is appropriate to keep a patient restrained lying flat on a bed. Hence, there is still room for improvement in terms of nurses’ knowledge on the application of restraint to avoid any physical harm of patients during restrictive interventions.

A study by [30] showed that over a decade ago only half of nurses would always try alternatives before applying restraints, while a high majority of nurses in this study would do so. We can interpret this as there being an increase in nurses’ intention to use alternative methods to physical restraints. On the other hand, despite the positive shift that has taken place regarding nurses’ attitudes towards alternative practices, the findings may reveal that psychiatric nurses perceived restraint as an inevitable practice in psychiatric inpatient settings, as safeguarding patient safety was their primary concern. Our results may also indicate that nurses have limited doubt or reflection about restraint practices [26], especially if nurses perceive physical restrictions as a usable intervention to maintain control over patients’ behavior [57], to support a peaceful ward environment [58] or to calm patients [32].

Nurses’ favorable attitudes toward patient physical restrictions might also explain why no statistically significant correlation was found between nurses’ attitudes and practices [11]. In addition, most nurses in our data had little emotional reaction to physical restraints. Scholars pointed out that emotional regulation among staff is a key element for successful de-escalation of patients’ aggression [59]. Over two decades ago, researchers found that education increases psychiatric nurses’ moral sensitivity [60]. Substantial evidence also suggests that continuing education can have a positive impact on nursing practice [60]. Further, a trial to test online education conducted in Finland showed that online training can decrease the duration of time patients are physically restricted on the ward. The key element of this online education was the nurses’ abilities to use their own self-reflection in conjunction with evidence-based knowledge [31].

Indeed, in our study, about one-third of the nurses reported not feeling bad if patients become more upset after being restrained. This finding may represent a lack of emotional reactions among nurses towards physical restraint, which is worrying. Previous studies have reported that ethical reflection among healthcare providers is key to the preservation of empathy [61], and that empathy among care staff is crucial to less restraint use [62]. On the other hand, if nurses are too emotional, having feelings such as guilt, it may lead to emotional exhaustion [63] or burnout [64]. In our data, only a few felt guilty about the application of restraint, and most did not. It has also been found that other strong emotions, such as anger, are associated with how nurses endorse restraint as an anger management technique for patients [65]. Therefore, based on our study results, it may be crucial to focus on nurses’ emotional reactions, not only on their knowledge levels, attitudes and practices regarding physical restrictions. However, this topic needs further research.

Our study only partially supports the assumption that knowledge, practices, and attitudes are correlated with each other. However, our study still shows that specific attitudes may indicate nurses’ rationales for why and how to use physical restraint in psychiatric inpatient care. For example, nurses may believe that being restrained is beneficial for patients. Our study may therefore indicate the importance of focusing more on nurses’ reflections and emotions using physical restraint on patients in psychiatric hospitals to ensure ethically sensitive care in psychiatric hospitals.

There are limitations in this study that need to be taken into consideration. First, the participants were working on psychiatric wards caring for patients with diverse mental health problems. Therefore, the study results may not be generalizable for other settings. Second, due to the high number of wards included in the study (8 in total) and the low number of nurses working in the hospital who responded to the survey (*N* = 98), we conducted a comparative analysis between the different wards. Therefore, the question of whether or not nurses’ knowledge, attitudes or practices vary depending on ward type remains unknown. This question deserves more investigation in the future. Third, quantitative methodology alone may not elicit in-depth information or human experience [66]. Fourth, we only looked at nurses’ perceptions towards practices, not objectively at what is really happening in practice. Fifth, correlations between knowledge, attitudes and practices can only describe associations between concepts, and therefore no causal relationship can be interpreted based on our results. Further, collecting data via questionnaires may be subject to social desirability response bias [67]. Finally, the data have been collected previously in 2016, which may cause doubts whether nurses’ responses are still the same. However, as there are no major changes conducted for the restraint-related care in the study hospital, and attitudes are changing slowly [68], the study results could still represent the current attitudes among nurses in Hong Kong. Despite these limitations, we assume that the results are usable for an international nursing audience to gain insight in enhancing nursing care related to the management of patient aggression.

Our findings show that, although the topic of physical restraint use is important in many countries, it may not be considered as important globally. Nurses in our study showed good knowledge levels about physical restraint, but more emphasis should be put on training methods to enhance nurses’ ethical sensitive thinking and to increase their competence in restraint use. For example, scenario-based stimulations, self-reflection, and role-playing could be employed. Further steps could be taken to emphasize restraint-related ethical issues. Through training, nurses may gain understanding on the psychological impact of the use of physical restraint and how it is experienced by patients [31]. Training for nurse managers would also be beneficial as it seems that their attitudes have an impact on the rates of containment used on the ward [69].

Trainings involving nurses and patients have been shown to effectively reduce restraint use [14]. In general, mental health hospitals should involve patients, relatives and staff in close collaboration and preventive activities for the emotional regulation and management of violence. Based on our results, one specific area of focus for future staff training could be relationships between family members and staff. As few nurses agreed that relatives have the right to refuse patients being restrained, our study reveals the limited degree to which nurses seem to be open to collaboration with patients and relatives to achieve joint decisions on anger management. A higher level of such a three-party collaboration is believed to be beneficial to patients’ emotional self-management [70] and a feeling of being safe [71], and hence could help identify alternatives to restraint. In addition, nurse managers could consider a blend of genders in the nursing staff, also on inpatient wards. Combining nurses of different ages and educational backgrounds, as well as both novice and senior nurses, could offer a more well-rounded shift team. The most important implication of our results may be the general foundation that, although the topic of physical restriction has been a research focus in many countries, there is still room to improve nurses’ awareness, knowledge and practices related to physical restraint on the local level.

## 5. Conclusions

This study shows that nurses had good restraint-related knowledge with satisfactory attitudes and practices. Our study also reveals some areas of misconceptions, and undesirable practices related to physical restraints. The findings suggest that psychiatric nurses perceived restraint as an inevitable practice in psychiatric inpatient settings, although no evidence of this can be found in the related scientific literature. Nurses may also have limited doubt or reflection regarding restraint practices. Future training should therefore be emphasized to enhance nurses’ ethical and sensitive thinking, to increase their competence in applying physical restraint as the last resort, and to work with patients and relatives collaboratively. Finally, thorough consideration in deciding a well-rounded shift team, regarding the diversity of various nursing attributes, may help to optimize care related to patient aggression.

## Figures and Tables

**Table 1:** Demographic characteristics of nurses (*n =* 98).

Attributes	*n*	Percentage
Gender		
Male	52	53.1
Female	46	46.9
Age (Divided by Median)		
<30	43	43.9
≥30	55	56.1
Years of Nursing Experience		
≤4	49	50
5–9	20	20.4
≥10	29	29.6
Education Level		
High school diploma or below	28	28.6
Bachelor’s degree	48	49
Master’s degree or above	22	22.4
Exposure to In-service Training for Management of Violence		
Yes	98	100

**Table 2:** Level of knowledge of restraints among psychiatric nurses (*n =* 98).

Concept Assessed by Each Item	No. of Nurses’ Correct Responses (%)
Safety vests or garments are or are not designed to prevent injury	68 (69.4)
Restraint is or is not legal when used for prevention of harm	83 (84.7)
* Restraint should or should not be used to closely observe patients	86 (87.8)
Patients are or are not allowed to refuse being restrained	51 (52)
Restraints require or do not require a physician’s order	88 (89.8)
* Confusion or disorientation is or is not the main purpose for restraints	79 (80.6)
Awake restrained patients should or do not need to be released every 2 h	** 26 (26.5)
* Restraints should or do not need to be applied snugly	** 25 (25.5)
Restraints should or should not be applied to patients lying flat on a bed	** 22 (22.6)
Restraints can or cannot damage skin and increase restlessness	71 (72.4)
Restraints should or do not need to be attached to side rails	77 (78.6)
* Sheet restraints may or may not be necessary at times	** 44 (44.9)
Nurses can or cannot be charged with assault for unnecessarily applying restraints	70 (71.4)
Restraint records do or do not need to be kept regularly	94 (95.9)
A physician’s order to restraining should or does not need to be specific	86 (87.8)
Nurses can or cannot legally restrain a patient upon emergency	77 (78.6)
* There are or are no good alternatives to restraints	73 (75.5)
Vest restraints are or are not linked to deaths	65 (66.3)

* Correct response should be “False” for this statement; ** Items with less than 50% correct responses.

**Table 3:** Comparison of the knowledge section according to nurses’ demographic characteristics.

Attributes	Min	IQR	Median	Max	*p*
Gender					
Male	3	3	12	16	0.05 ^†^
Female	3	3	11	17	
Age (Divided by Median)					
<30	3	6	11	17	** 0.001 ^†^
≥30	8	3	12	16	
Years of Nursing Experience					
≤4	3	5	11	16	* 0.003 ^‡^
5–9	10	4	13	17	
≥10	8	3	12	16	
Education Level					
High School Diploma or below	3	3	11	16	** 0.001 ^‡^
Bachelor’s degree	3	4	12	16	
Master’s degree or above	8	2	14	17	

^†^—Mann–Whitney U Test; ^‡^—Kruskal–Wallis Test; * *p* ≤ 0.05; ** *p* ≤ 0.001.

**Table 4:** Nursing practice issues regarding physical restraints (*n =* 98).

Items	No. of Nurses’ Responses (Percentage)		
	Always	Sometimes	Never
Trying alternative measures before applying restraints	93 (94.9)	5 (5.1)	0
Identifying the reason of unacceptable behavior before using restraints	74 (75.5)	24 (24.5)	0
Making counter-suggestions to a doctor for unnecessary restraints	54 (55.1)	37 (37.8)	7 (7.1)
Answering restrained patients’ calls for help as soon as possible	88 (89.8)	7 (7.1)	3 (3.1)
Reviewing restraint conditions every 2 h	86 (87.8)	7 (7.1)	5 (5.1)
Checking skin condition while providing care to restrained patients	92 (93.9)	5 (5.1)	1 (1.0)
Explaining the reason of restraints to patients	87 (88.8)	11 (11.2)	0
Explaining the reason of restraints to relatives	71 (72.4)	27 (27.6)	0
Telling patients the removal time of restraints	82 (83.7)	15 (15.3)	1 (1.0)
Telling relatives the removal time of restraints	56 (57.1)	38 (38.8)	4 (4.1)
* Restraint is necessary in acute care settings.	69 (70.4)	22 (22.4)	7 (7.1)
* Routinely applying restraints to disoriented acute care patients	8 (8.2)	58 (59.1)	32 (32.7)
* Routinely applying restraints to patients with confusion and IV lines	9 (9.2)	58 (59.2)	31 (31.6)
Reading the hospital’s restraint policy	82 (83.7)	13 (13.3)	3 (3.1)
* Applying more restraints during periods of reduced staff	15 (15.3)	44 (44.9)	39 (39.8)
Staff members work together to identify alternatives to restraints.	59 (60.2)	39 (39.8)	0
A restraint device is available in the unit when needed.	88 (89.8)	10 (10.2)	0
Sedating patients with medications rather than applying restraints.	62 (63.3)	36 (36.7)	0

* Negatively phrased statements (unfavorable practice).

**Table 5:** Comparison of practice section according to nurses’ demographic characteristics.

Attributes	Min	IQR	Median	Max	*p*
Gender					
Male	41	5	48	52	* 0.042 ^†^
Female	38	4	46	53	
Age (Divided by Median)					
<30	38	15	46	53	0.285 ^†^
≥30	41	3	47	52	
Years of Nursing Experience					
≤4	38	4	46	52	0.313 ^‡^
5–9	41	4	47	53	
≥10	43	4	46	51	
Education Level					
High School Diploma or below	41	6	46	51	0.140 ^‡^
Bachelor’s degree	38	3	47	52	
Master’s degree or above	43	5	46	53	

^†^—Mann–Whitney U Test; ^‡^—Kruskal–Wallis Test; * *p* ≤ 0.05.

**Table 6:** Nursing attitudes towards the use of restraints (*n =* 98).

Items	No. of Nurses’ Responses (Percentage)		
	Agree	Neutral	Disagree
Family members have the right to refuse restraints.	25 (25.5)	36 (36.7)	37 (37.8)
Nurses have the right to refuse restraints.	69 (70.4)	19 (19.4)	10 (10.2)
Patients have the right to refuse restraints.	59 (60.2)	19 (19.4)	20 (20.4)
Feeling guilty about application of restraints.	13 (13.3)	28 (28.6)	57 (58.2)
* Restraints are used because of reduced staffing.	14 (14.3)	22 (22.4)	62 (63.3)
Feeling embarrassed if use of restraint is discovered by relatives.	55 (56.1)	24 (24.5)	19 (19.4)
* Hospital is legally responsible to protect patients by using restraints.	78 (79.6)	9 (9.2)	11 (11.2)
Feeling bad if patients become more upset after being restrained.	31 (31.6)	37 (37.8)	30 (30.6)
It is more important to let the patients know nurses care about them.	86 (87.8)	7 (7.1)	5 (5.1)
Patients’ confusion level may be increased after being restrained.	15 (15.3)	21 (21.4)	62 (63.3)
Patients suffer a loss of dignity because of restraints.	31 (31.6)	41 (41.8)	26 (26.5)
Feeling knowledgeable about caring for restrained patients.	88 (89.8)	9 (9.2)	1 (1.0)

* Negatively phrased statements (unfavorable attitudes).

**Table 7:** Comparison of attitudes section according to nurses’ demographic characteristics.

Attributes	Min	Mean	SD	Median	Max	*p*
Gender						
Male	18	25.33	3.30	25	34	0.114 ^†^
Female	20	26.48	3.85	26	36	
Age (Divided by Median)						
<30	18	25.79	3.86	26	36	0.853 ^†^
≥30	19	25.93	3.40	27	33	
Years of Nursing Experience						
≤4	18	25.27	3.18	25	32	0.232 ^‡^
5–9	20	26.20	4.35	27.5	36	
≥10	19	26.66	3.64	27	33	
Education Level						
High school diploma or below	18	24.89	2.90	25	31	* <0.001 ^‡^
Bachelor’s degree	20	25.19	3.40	26	32	
Master’s degree or above	22	28.59	3.61	28	36	

^†^—*t*-test; ^‡^—ANOVA; * *p* ≤ 0.001.

**Table 8:** Correlations (Spearman’s rho) between the total scores of knowledge level, practice and attitudes.

Study variables	Knowledge	Practice	Attitudes
Knowledge	-		
Practice	0.38 *	-	
Attitudes	0.37	0.28	

* *p* ≤ 0.001.

## Data Availability

The data presented in this study are available on request from the corresponding author. The data are not publicly available due to ethical and privacy reasons.
